# Experimentally designed electrochemical sensor for therapeutic drug monitoring of Ondansetron co-administered with chemotherapeutic drugs

**DOI:** 10.1186/s13065-022-00871-5

**Published:** 2022-10-13

**Authors:** Mona A. Abdel Rahman, Shimaa A. Atty, Sally S. El-Mosallamy, Mohamed R. Elghobashy, Hala E. Zaazaa, Ahmed S. Saad

**Affiliations:** 1 Analytical Chemistry Department, Faculty of Pharmacy, October 6 University, 6 October City, PO box 12858, Giza, Egypt; 2 Pharmaceutical Chemistry Department, Egyptian Drug Authority, 51 Wezaret El-Zeraa St, Cairo, Egypt; 3 Analytical Chemistry Department, Faculty of Pharmacy, Cairo University, Kasr El-Aini St, PO 11562, Cairo, Egypt; 4 Medicinal Chemistry Department, PharmD program, Egypt-Japan University of Science and Technology (E-JUST), New Borg El-Arab City, PO 21934, Alexandria, Egypt

**Keywords:** Design of experiment, Potentiometric sensor, Ondansetron assay, Therapeutic drug monitoring, Plasma

## Abstract

The experimental design extracts valuable information about the main effects and interactions from the least number of experiments. The current work constructs a solid-state sensor for selective assay of Ondansetron (OND) in pharmaceutical dosage form and plasma samples. During optimization, the Design Expert^®^ statistical package constructed a custom design of 15 sensors with different recipes. We fed the software with the experimentally observed performance parameters for each sensor (slope, LOQ, correlation coefficient, and selectivity coefficient for sodium ions). The computer software analyzed the results to construct a prediction model for each response. The desirability function was adjusted to optimize the Nernstian slope, minimize the LOQ and selectivity coefficients, and maximize the correlation coefficient (r). The practical responses of the optimized sensor were close to those predicted by the model (slope = 60.23 mV/decade slope, LOQ = 9.09 × 10^–6^ M, r = 0.999, sodium selectivity coefficient = 1.09 × 10^−3^). The sensor successfully recovered OND spiked to tablets and human plasma samples with mean percentage recoveries of 100.01 ± 1.082 and 98.26 ± 2.227, respectively. Results were statistically comparable to those obtained by the reference chromatographic method. The validated potentiometric method can be used for fast and direct therapeutic drug monitoring of OND co-administered with chemotherapeutic drugs in plasma samples.

## Introduction

With rising global competitiveness and the expanding effect of information technology, the pharmaceutical sector urgently needs to enhance its operational efficiency and increase product quality. Pharmaceutical companies restructured and standardized the Quality-by-Design (QbD) approach to satisfy customer needs and achieve quality in the operation process and excellence in the market [1]. In the pharmaceutical industry, the QbD is a systematic approach that defines objectives and understands the sources of variability to control the product and process. In its Q8 and Q11guidelines, the International Conference on Harmonization (ICH) has recently mentioned the QbD and its application in the pharmaceutical industry. The QbD in analytical method development —also known as Analytical Quality by Design (AQbD)—represents a leap that achieves the desired method performance. In a few words, the QbD's primary focus is to maximize productivity and quality and minimize risk. The design of experiments (DoE) is a vital part of QbD. DoE uses a limited number of experiments to identify the critical method variables based on statistical significance testing. It determines the optimum range for each variable to achieve the desired analytical performance with acceptable robustness [2–4]. Cancer is the uncontrollable growth and dissemination of abnormal cells throughout the body [5]. Cancer is the world's second most common cause of death, killing more than 8 million people annually; cancer incidence is predicted to rise by more than 50% in the coming decades [6, 7].

Chemotherapeutic agents are used individually or in combination to treat different cancer types. However, it risks emetogenic side effects such as nausea and vomiting [8]. The control of such side effects is crucial for effective treatment and patient compliance. Ondansetron (OND), a type 3 serotonin (5-HT3) receptor antagonist in the brain and GIT, is the first-line treatment for the emetogenic side effects of chemotherapy [9]. OND is chemically known as (RS)-9-methyl-3-[(2-methyl-1H-imidazole-1-yl) methyl]-2,3-dihydro-1H-carbazol-4(9H)-one. It is a basic compound (p*K*_a_ of 7.34) highly soluble in acidic media and sparingly solubility in water [10]. It treats nausea and vomiting during chemotherapy, radiotherapy, anxiolytic, and neuroleptic problems. The 5-HT3 released during chemotherapy, radiotherapy, and surgery stimulate the 5-HT3 receptors in the chemoreceptor trigger zone that sends signals to the vomiting center in the brain [11–13].

Cytochrome P-450 enzymes (including the polymorphic CYP2D6 and CYP1A2) are involved in the biotransformation of OND, leading to a significant intra-individual variation in OND plasma concentrations and thus its efficacy [14]. Therefore, it was mandatory to develop a real-time analytical method for the rapid, sensitive, and selective assay of OND to adjust its dose during chemotherapy.

British Pharmacopoeia reports non-aqueous titrimetric and HPLC methods [15] for OND. The literature reported additional spectrofluorimetry [16], spectrophotometry [17–23], voltammetry [24, 25], radioimmunoassay [26], flow injection [27, 28], thin-layer chromatography [29, 30], high-performance liquid chromatography [31–41], and liquid chromatography-tandem mass spectrometry (LC–MS/MS) [42, 43]. The discussed methods come at a high expense and call for laboratories that are well-equipped. Thus, the aforementioned techniques did not fit routine analysis because they involved a lengthy analysis time, a complex instrument setup, and multiple manipulation steps. Ion-selective electrode-potentiometry is a low-cost, economical, simple-to-use, and non-destructive technique. Ion-selective electrodes (ISEs) enable continuous monitoring of minimal sample volume without sample pretreatment. They are also suitable for colored or cloudy samples [44, 45]. A single potentiometric method was reported for OND determination [46]. However, the current OND sensor was the first to use the QbD approach to reach the target analytical response.

The current work aims to develop an ion-selective electrode potentiometric sensor for the therapeutic drug monitoring of OND in plasma samples and routine analysis in quality control laboratories. The mentioned objective demands a rapid, sensitive, and selective sensor response with minimal sample treatment. The sensor development followed a QbD approach to reach the desired analytical goal. Based on our recently published work [47], we identified the critical sensor variables and the response parameters that have to be monitored to achieve the desired analytical objective.

## Experimental

### Apparatus

Potential measurements were performed utilizing a digital potentiometer, Jenway model 3330 (Essex, UK), with a double junction Ag/AgCl reference electrode, Orion, ThermoScientific no. 900200, and a magnetic stirrer, Bandelin Sonoro, R510S (Budapest, Hungary), and a Jenway (924051, UK) glass electrode were used.

### Reagents, reference standards and materials

Analytical-grade chemicals were employed in this study. Poly(vinyl)chloride (PVC), Nitrophenyl octyl ether (NPOE), and dioctyl phthalate (DOP) were purchased from Alfa-Aesar (Ward Hill, MA, USA). Phosphotungstic acid (PT), phosphomolybdic acid (PM), sodium tetraphenylborate (TPB), tetrakis (TKS), ammonium Reineckate (RK), carboxymethyl-β-cyclodextrin (CMBCD), hydroxypropyl- β- cyclodextrin (HPBCD), calix-[8]-arene (CX_8_), calix-[4]-arene (CX_4_), and tetrahydrofuran (THF) were purchased from Aldrich (Steinheim, Germany). Potassium dihydrogen phosphate, sodium hydroxide, hydrochloric acid, and metal salts were purchased from El-NASR Pharmaceutical Chemical Co., Abu-Zabaal, Cairo, Egypt, as chlorides. Phosphate buffer pH 5.5 was prepared by adjusting the pH of 1 × 10^−3^ M potassium dihydrogen phosphate solution (using bi-distilled water as solvent) to pH 5.5 with 1 M NaOH solutions. Ondansetron working standard (99.87% purity) was obtained from Sigma-Aldrich (St. Louis, MO, USA). Zofran^®^ tablet (Batch no: B74319 C) containing 8.0 mg OND; was purchased from the local market. Plasma samples were obtained from Vacsera Co. (Giza, Egypt).

Stock standard solution (1.0 × 10^−2^ M) OND was prepared in phosphate buffer pH 5.5. Serial dilutions of the stock standard solution were carried out to prepare OND working standard solutions in the range 1.00 × 10^−6^ to 1.0 × 10^−3^ M using phosphate buffer pH 5.5 as a solvent.

### Procedure

#### Optimization of the PVC membrane composition

##### Custom experimental design

We adopted a custom experimental design that included three categorical factors: plasticizer, ion exchanger, and ionophore types. The design included 15 sensors with two levels for the plasticizer type (NPOE and DOP), five levels for the ion-exchanger type (TPB, PT, PM, TKS, RK). and five levels for the ionophore types (BCD, HPBCD, CMBCD, CX_4_, CX_8_) levels were coded as shown in Table 1.

**Table 1:** The custom experimental design architecture for the levels and components of the studied sensors and the observed responses for each sensor

Sensor no	Factor 1		Factor 2		Factor 3		Response 1	Response 2	Response 3	Response 4
	Plasticizer		Ion exchanger		Ionophore		Slope	r	pLOQ	$\mathrm{log}{K}_{OND, {Na}^{+}}^{pot}$
1	−1	NPOE	2	RK	−2	BCD	53.55	0.9984	4.57	−2.50
2	1	DOP	2	RK	−1	HPBCD	53.10	0.9992	4.57	−3.71
3	−1	NPOE	−1	PT	−1	HPBCD	62.79	0.9983	4.36	−4.22
4	−1	NPOE	1	TKS	1	CX4	59.70	0.9972	5.32	−2.96
5	1	DOP	−1	PT	0	CMBCD	62.56	0.9986	4.79	−3.44
6	1	DOP	1	TKS	2	CX8	56.49	0.9976	5.03	−3.56
7	−1	NPOE	0	PM	−1	HPBCD	59.05	0.9980	4.01	−4.23
8	−1	NPOE	−2	TPB	2	CX8	59.05	0.9965	4.57	−2.11
9	−1	NPOE	−1	PT	−2	BCD	66.41	0.9988	5.04	−2.88
10	1	DOP	−1	TPB	−2	BCD	60.78	0.9960	5.04	−2.86
11	−1	NPOE	2	RK	2	CX8	59.05	0.9981	4.57	−2.96
12	−1	NPOE	0	PM	0	CMBCD	56.04	0.9994	4.52	−3.12
13	−1	NPOE	1	TKS	0	CMBCD	60.15	0.9980	5.04	−2.94
14	−1	NPOE	−2	TPB	1	CX4	66.75	0.9971	5.32	−2.25
15	1	DOP	0	PM	1	CX4	50.25	0.9986	4.97	−3.26

The slope (S), correlation coefficient (r), the negative logarithm of the quantification limit (pLOQ), and the selectivity coefficient for sodium ions ($\mathrm{log}{K}_{OND, Na}^{pot}$) were recorded for each of the 15 sensors (Table 1). The experiments evaluated the main effects and interactions between the three studied factors. We adjusted the desirability function to achieve the optimum slope and maximum pLOQ and r, and minimum $\mathrm{log}{K}_{OND, Na}^{pot}$.

##### Membrane fabrication and sensor assembly

Solutions of the membrane components were separately prepared by transferring accurate weights of each component, including PVC, plasticizer (NPOE and DOP), ion-exchanger (TPB, PT, PM, TKS, RK), and ionophore (BCD, HPBCD, CMBCD, CX_4_, CX_8_) in a tube and dissolve it in an adequate amount of THF. The membrane recipes were prepared by mixing accurate volumes of the prepared solutions as described in Table 1. A micropipette was used to drop cast an accurate volume of the membrane component over the coated glassy carbon electrode surface electrochemically coated with polyaniline [48], then left to dry at room temperature.

##### Sensor calibration

The potential differences were measured between the working and the reference electrode immersed in OND solutions (1.00 × 10^−6^ to 1.0 × 10^−2^ M) and stirred at 100 rpm until a stable response was reached within ± 1.0 mV. The sensor was then washed with distilled water before measuring the potential differences. Similarly, calibrations were held in human plasma samples after ten-fold dilution with phosphate buffer. The regression equations were computed for each sensor by plotting the potential against the logarithm of the molar concentration. Slopes, correlation coefficients, and limits of quantification were calculated.

##### Effect of pH

The optimized sensor was used to monitor the difference in potential when pH is deliberately changed in 1.0 × 10^−2^ M and 1.0 × 10^−3^ M solutions of OND. The change in pH was done by adding a small amount of aqueous 0.1 M NaOH and 0.1 M HCl. The potential and pH were recorded after each addition and plotted against one another to obtain the pH curve.

##### Stability of the proposed sensor

The potential difference between the optimized sensor and the reference electrode was recorded in OND solutions (2.68 × 10^−5^–4.07 × 10^−4^ M). The time required to reach a stable response within one mV from the equilibrium potential was recorded.

##### Sensor selectivity

The IUPAC's Separate Solutions Method (SSM) [49] was used to calculate the potentiometric selectivity coefficient ${K}_{OND, Int}^{pot}$ for different cationic contaminants (K^+^, Na^+^, NH_4_^+^, Ca^2+^, Mg^2+^, Cd^2+^, Fe^2+^, and Cr^3+^). Measurements were performed in 1.0 × 10^−3^ M solutions of OND and the interfering cations, and the selectivity coefficient was calculated according to the Nicol sky–Eisenman equation [50].

1 $$\mathrm{log}{k}_{OND, Int}^{pot}=\frac{{E}_{Int}-{E}_{OND}}{S}+(1-\frac{{Z}_{OND}}{{Z}_{Int}})\mathrm{log}{a}_{OND}$$

where ${K}_{OND, Int}^{Pot}$ the selectivity coefficient, ${E}_{OND}$ and ${E}_{Int}$ represent the potential of OND and interfering ions, respectively, ${z}_{OND}$ and ${z}_{Int}$ refer to the charge of OND and the interfering ions, respectively, S is the calibration curve slope, and ${a}_{OND}$ the OND concentration.

##### Sensor reversibility

The reproducibility was evaluated by reciprocally dipping the optimized sensor and the reference electrode in two different OND solutions (1.0 × 10^–4^ M and 1.0 × 10^–3^ M).

#### Application

##### Assay of OND in Zofran^®^ tablets

The average weight of one Zofran^®^ tablet was determined from the uniform powder of five crushed tablets. The powder was transferred into a 50 mL measuring flask and sonicated with 30 mL phosphate buffer (pH 5.5) for 15.0 min, and the volume was then completed using the same solvent. The solution was diluted ten times before measuring the potential difference between the optimized sensor and the reference electrode before and after adding 1 mL of OND stock standard solution 1 × 10^−2^ M. The sample concentration (C_x_) was calculated from the standard addition equation [51]:

2 $$\mathrm{Cx}=\mathrm{Cs}\left(\frac{\mathrm{Vs}}{\mathrm{Vx}+\mathrm{Vs}}\right){\left[{10}^{n(\Delta E/S)}-\frac{\mathrm{Vx}}{\mathrm{Vs}+\mathrm{Vx}}\right]}^{-1}$$

where C_x_ is the concentration in the sample solution, V_x_ is the sample solution volume, Cs and Vs are the concentration and volume, respectively, of added standard solution, ΔE is the change in potential after standard addition, and S stands for the slope of the calibration curve in mV per decade.

##### Assay of spiked plasma samples

Human plasma samples (450 µL) were transferred into a 5 mL measuring flask and spiked with 50 µL of OND standard solutions (1.0 × 10^−2^–1.0 × 10^−3^ M). The volume was completed to the mark using the phosphate buffer. The previously mentioned standard addition technique was followed to determine OND concentration in the spiked plasma samples.

## Results and discussion

The quality by design approach was applied to develop a potentiometric method that fits for definite analytical purposes. The study employed the design expert program to (1) develop a custom experimental design, (2) analyze the results to evaluate the effect of the studied factors, and (3) build a prediction model that serves to (4) optimize the sensor composition according to the desired analytical performance and intended analytical purposes.

### Optimization study

The sensor performance is a function of its composition and assembly; a properly selected membrane recipe will inevitably lead to the desired performance [52]. We developed an optimization study to evaluate the significance and quantified the main effects and interactions among the studied factors (plasticizer, ion exchanger, and ionophore) (Table 1). The study included two plasticizers (NPOE, DOP), five ion exchangers (TPB, PT, PM, TKS, RK), and five ionophores (BCD, HPBCD, CMBCD, CX_4_, CX_8_). The measured outcomes included the slope, correlation coefficient, quantification limit, and sodium selectivity as descriptors for the method performance; the results were statistically analyzed using One-Way ANOVA (Table 2).

**Table 2:** The One-way ANOVA analysis of the estimated performance parameters

	Source	Sum of squares	df	Mean square	F-value	p-value	
A.Slope	Model	234.84	5	46.97	6.19	0.0093	Significant^a^
	A-PS	43.63	1	43.63	5.75	0.0400	
	B-IE	191.20	4	47.80	6.30	0.0106	
	Residual	68.26	9	7.58			
	Cor Total	303.10	14				
B.Correlation Coefficient	Model	0.0000	4	2.548E-06	9.48	0.0020	Significant^a^
	B-IE	0.0000	4	2.548E-06	9.48	0.0020	
	Residual	2.687E-06	10	2.687E-07			
	Cor Total	0.0000	14				
C.pLOQ	Model	1.76	9	0.1950	6.83	0.0239	Significant^a^
	A-PS	0.0730	1	0.0730	2.56	0.1708	
	B-IE	0.4495	4	0.1124	3.93	0.0827	
	C-IP	0.8236	4	0.2059	7.21	0.0263	
	Residual	0.1428	5	0.0286			
	Cor Total	1.90	14				
D.$\mathrm{log}{K}_{OND, {Na}^{+}}^{pot}$	Model	3.48	4	0.8693	4.35	0.0270	Significant^a^
	C-IP	3.48	4	0.8693	4.35	0.0270	
	Residual	2.00	10	0.1996			
	Cor Total	5.47	14				

^a^ at significance level α = 0.05

We measured outcomes that serve the desired analytical objective. An ideal sensor produces a stable Nernstian slope, maximum correlation coefficient, minimal quantification limit, and minimal sodium selectivity coefficient. Finally, we used the prediction model to select the levels of each factor that can achieve the defined method objective. The defined outcomes were calculated according to the IUPAC recommendations [49].

The Design Expert^®^ was fed with the practically estimated outcomes for each studied sensor. The program analyzed each of the four responses to build a separate prediction model for each. The prediction models were statistically analyzed using the One-way ANOVA test to determine the significance of the prediction model and the factors that significantly affect each outcome. We decided to use the pLOQ instead of the LOQ directly to minimize decimal places. Therefore, it is desired to maximize the pLOQ for better sensor performance. One-way ANOVA proved the ability of the model to predict the sensor slope, correlation coefficient, pLOQ, and sodium selectivity (p < 0.05).

We used the One-Way ANOVA to estimate the factors that significantly affect the prediction of each of the estimated outcomes. The plasticizer (p = 0.0400) and ion-exchanger (p = 0.0106) significantly affect the slope prediction model. While the type of ion exchanger used (p = 0.0020) significantly affects the correlation coefficient prediction model. The ionophore (p = 0.0263) significantly influences the pLOQ prediction model. The ionophore (p = 0.0270) significantly affects the sodium selectivity prediction model, (Table 2).

We studied the plasticizer at two different levels (NPOE and DOP). ANOVA results proved a significant effect for the type of plasticizer on the slope. The plasticizer determines membrane properties such as polarity and conductivity. In addition, it regulates membrane conductivity by controlling the mobility of the membrane components. It modulates the membrane polarity, thus affecting selectivity and partitioning at the membrane solution interface, exudation of membrane components, and deposition of plasma proteins [53].

We studied five different levels of the ion-exchanger (TPB, PT, PM, TKS, RK). The ANOVA test proved the ion exchanger type’s significant effect on the slope and correlation coefficient (Table 2). The ion exchanger modifies the membrane properties and affects the analyte exchange at the membrane solution interface. The lipophilic ion exchanger controls the membrane conductivity, prevents co-extraction of similarly charged ions (Donnan exclusion effect), and selectively extracts the analyte ion from the sample solution. Its lipophilic nature minimizes loss due to leaching into the sample solution [54].

Ionophores are lipophilic molecules that bind selectively and reversibly to the analyte ion. It prevents efflux of the analyte ion and keeps its concentration constant within the membrane matrix. This minimizes potential perturbations and restricts potential changes to the change in the sample solution concentration [55].

We analyzed the results at five different ionophore levels (BCD, HPBCD, CMBCD, CX_4_, CX_8_). The ionophore type significantly affected the quantification limit and selectivity for sodium ions. Ionophores selectively recognize and reversibly bind the OND within the membrane matrix. Thus ionophores are lipophilic molecules that bind selectively and reversibly to the analyte ion. It prevents efflux of the analyte ion and keeps its concentration constant within the membrane matrix. This minimizes potential perturbations and restricts potential changes to the change in the sample solution concentration [51].

**Fig. 1:**
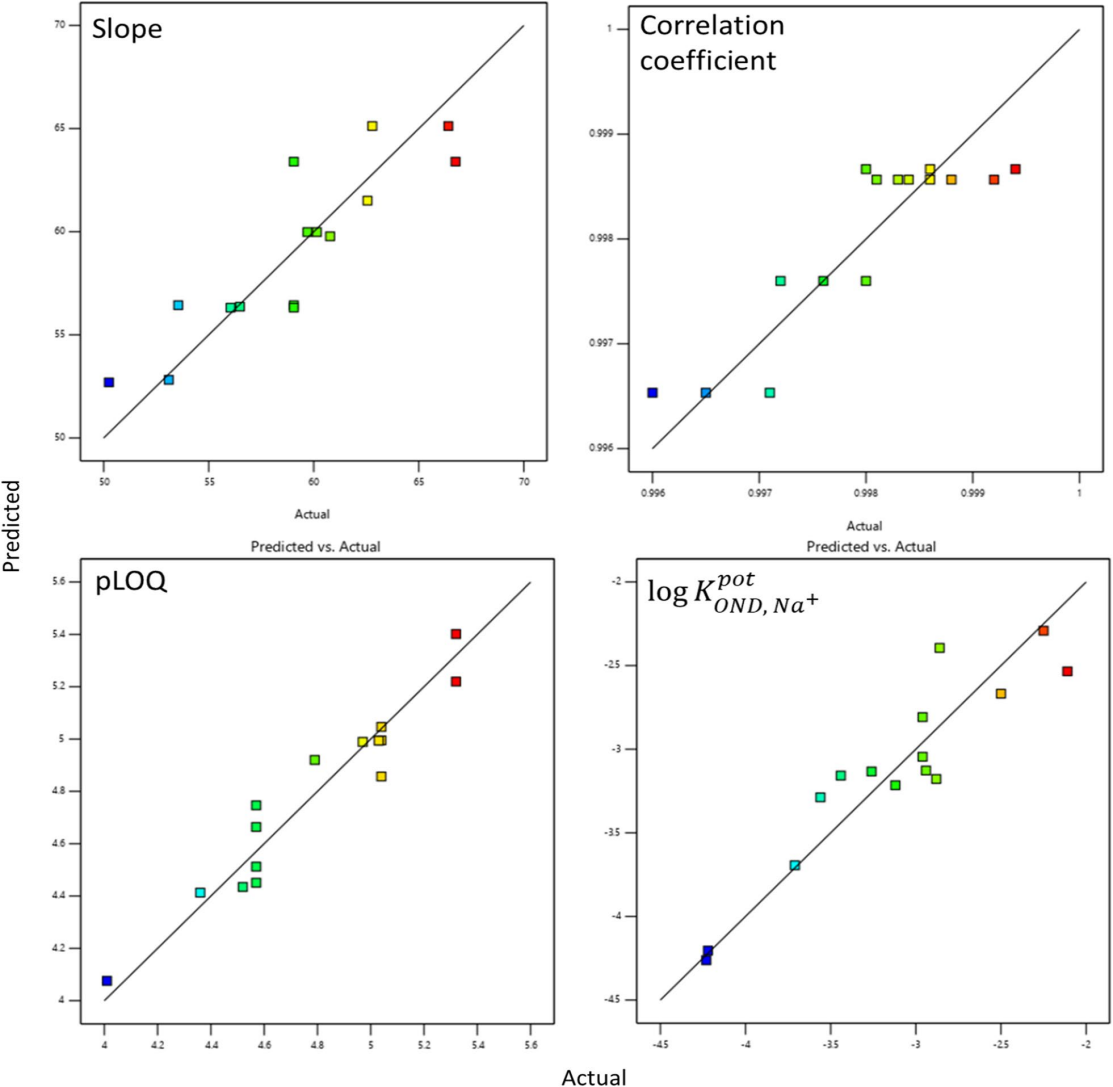
Predicted versus actual values of the slope, correlation coefficient, pLOQ, and $\mathrm{log}{K}_{OND, {Na}^{+}}^{pot}$ for the studied sensors

The program constructed four prediction models (one for each outcome). The predicted values were close to the actual values [56, 57], as shown in Fig. 1.

**Fig. 2:**
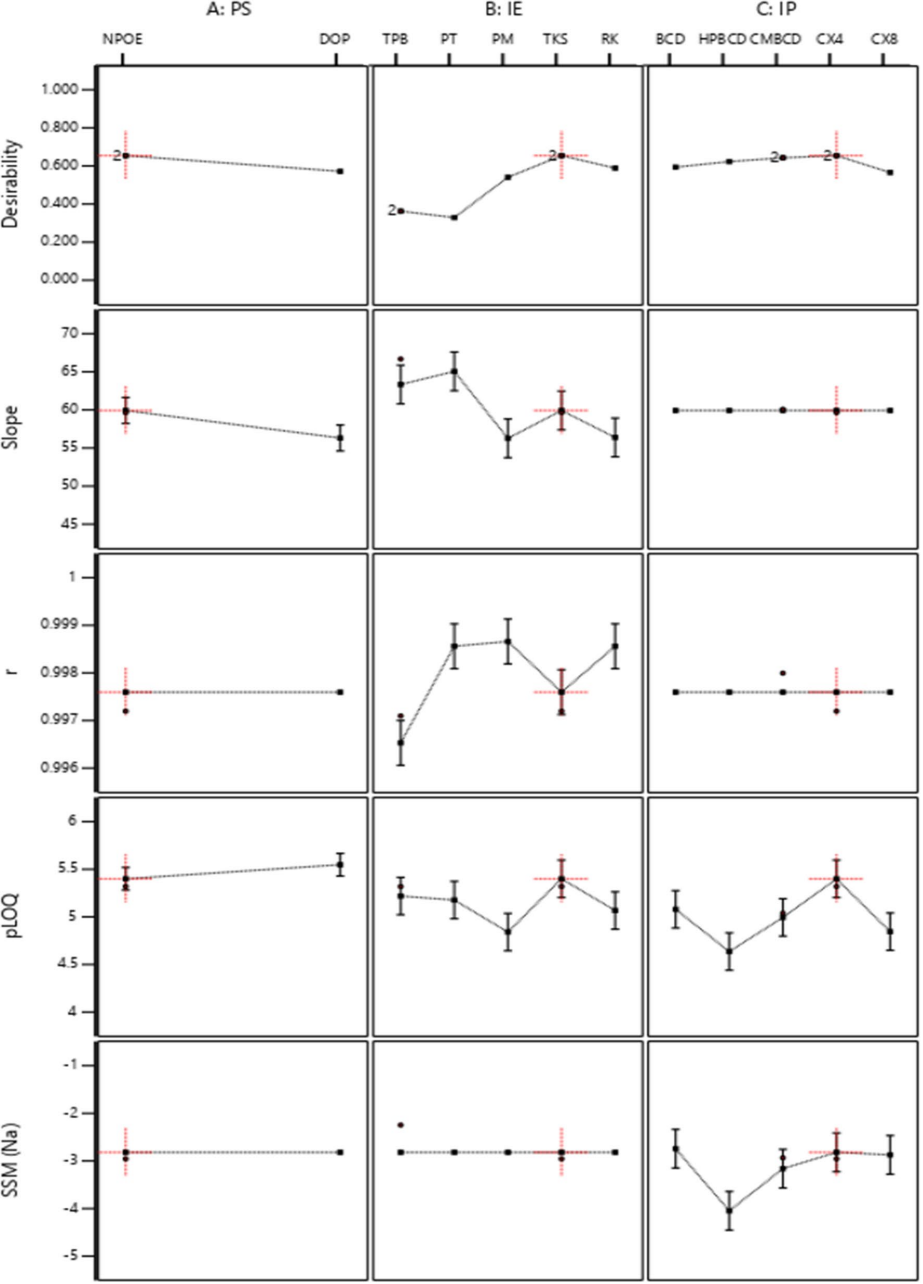
Results of the desirability function for the optimization of the sensor composition to achieve the desired optimum slope, maximum correlation coefficient, maximum pLOQ, and minimum $\mathrm{log}{K}_{OND, {Na}^{+}}^{pot}$

**Fig. 3:**
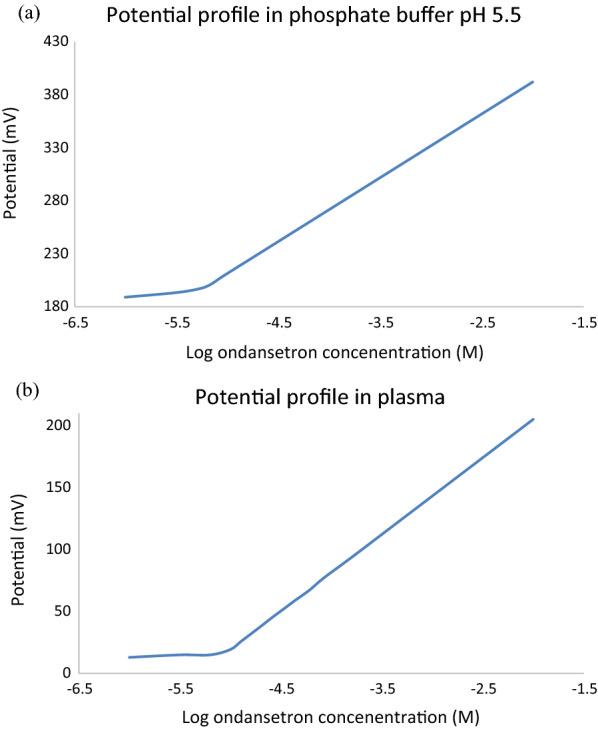
The potential profile for the optimized sensor in **a** phosphate buffer pH 5.5 and **b** plasma diluted (1:10) with phosphate buffer pH 5.5

The desirability function used the built models to determine the optimal membrane composition according to the desired outcomes. We claimed a Nernstian slope, maximum correlation coefficient, maximum pLOQ, and minimum sodium selectivity coefficient ($\mathrm{log}{K}_{OND, {Na}^{+}}^{pot})$. The desirability function suggested the optimum membrane recipe. The recipe included NPOE as a plasticizer, TKS as an ion exchanger, and CX_4_ as an ionophore (Fig. 2). We constructed the suggested sensor and plotted the calibration graphs (Fig. 3). We compared the theoretical response parameters calculated by the software (slope = 59.99, r = 0.999, pLOQ = 5.401, $\mathrm{log}{K}_{OND, Na}^{pot}$=-2.96) with those obtained practically (slope = 60.23, r = 0.999 and pLOQ = 5.401, and $\mathrm{log}{K}_{OND, Na}^{pot}$= -2.96) to verify the model; the results were interestingly comparable.

### Response characteristics of the optimized sensor

We followed the ICH guidelines [58] to validate the developed method (Accuracy, selectivity, precision etc.), as shown in Table 3. Whereas, the potentiometric sensor response parameters were evaluated according to the IUPAC guidelines [49], as shown in Table 3. The slope was calculated for the linear part of the potential-logarithm concentration relationship (Fig. 3). The optimized sensor displayed a Nernstian slope in phosphate buffer (60.23 mV/decade) and diluted plasma samples (61.90 mV/decade), as shown in Table 3. The detection limit, quantification limi,correlation coefficient, and linear range are shown in Table 3.

**Table 3:** Validation parameters of the optimized sensor

Parameter	In phosphate buffer pH 5.5	In plasma
Linearity		
Slope (mV per decade)	60.23	61.90
Correlation coefficient (r)	0.999	0.998
Concentration range (M)	9.09 × 10^−6^–1.0 × 10^−2^	1.0 × 10^−5^‒1.0 × 10^−2^
LOQ (M)	9.09 × 10^−6^	1.00 × 10^−5^
LOD (M)	4.24 × 10^−6^	7.91 × 10^−6^
Accuracy (mean ± SD)	100.05 ± 0.577	100.15 ± 0.516
Precision		
Repeatability (%RSD)	1.051	1.970
Intermediate precision (%RSD)	1.106	1.889
Working pH range	2‒6	
Response time (s)	3–6	
Life span (weeks)	4	

**Fig. 4:**
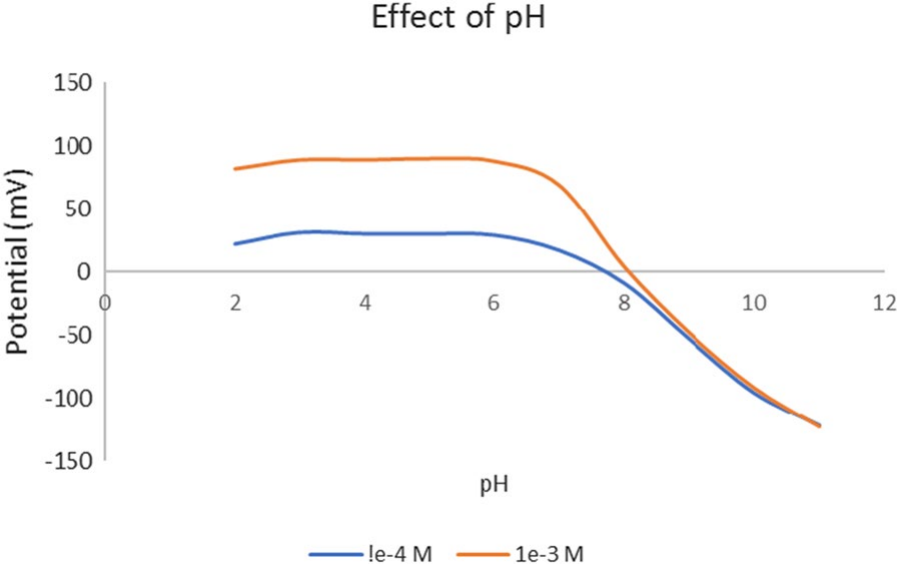
The effects of pH on the potential response of the optimized sensor

The characteristics of the electrochemical response and validation parameters of OND analysis are shown in Table 3. The method's accuracy, repeatability, and intermediate precision were assessed at three different concentrations (1.0 × 10^−4^, 5.0 × 10^−4^, and 1.0 × 10^−3^ M) in phosphate buffer pH 5.5 and diluted plasma, as shown in Table 3. The sensor proved a stable potential response in pH 2–6, as shown in Table 3 and Fig. 4.

**Fig. 5:**
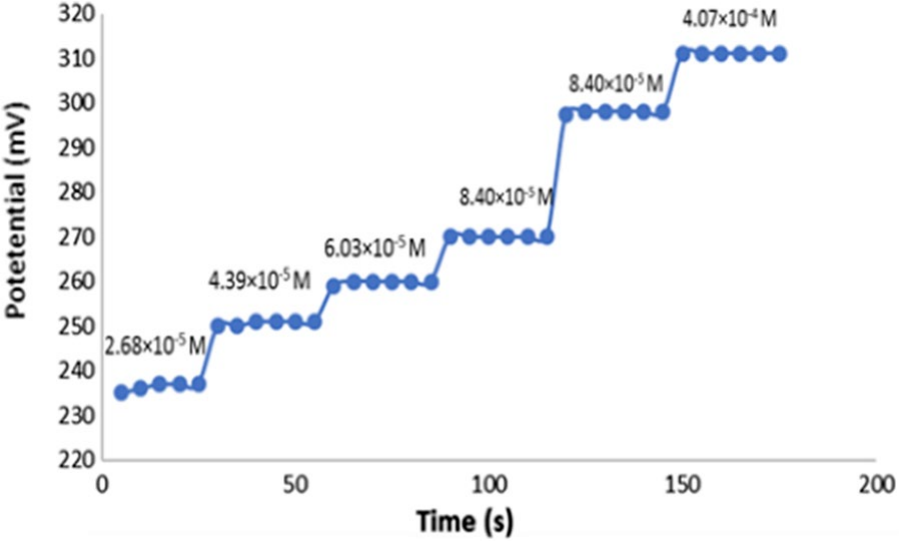
Dynamic response profile in successively increasing Ondansetron concentrations (2.68 × 10^−5^ M to 4.07 × 10^−4^ M) pH 5.5

Below pH 6, the potential declined steeply. The decrease in the potential is attributed to a corresponding reduction in the charged OND species as the pH increases above its pKa value (7.34), and OND converts to the free basic form (uncharged). The optimized sensor equilibrates rapidly across the entire concentrations range resulting in a fast and stable response, as shown in Fig. 5. In addition, the sensor achieved more rapid equilibrium at higher OND concentrations (3 s) than at lower concentrations (6 s), as shown in Fig. 5 and Table 3.

The sensor proved a stable slope ± 1.0 mV/decade for 4 weeks (Table 3). The loss in response is attributed to the leaching of membrane components from the PVC membrane.We calculated the quantification and detection limits according to the IUPAC recommendation. Results proved the sensor's capacity to detect OND in highly diluted solutions down to 9.09 × 10^–6^ M in phosphate buffer and 1.00 × 10^–5^ in human plasma (Table 3).

**Fig. 6:**
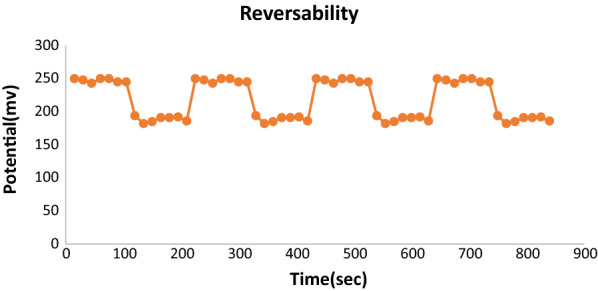
The effects of reversibility on the optimized sensor at low (1.0 × 10^−4^ M) to high (1.0 × 10^−3^ M) Ondansetron solutions

The performance of the suggested sensor may be utilized for 4 weeks without significantly changing. The reproducibility was evaluated by assessment of the reversibility of the sensor; the reversibility study expressed the fast OND exchange at the sensor sample interface, as shown in Fig. 6.

The selectivity study performed using the separate solution method [59], expressed the sensor selectivity for OND in the presence of commonly interfering cationic species naturally present in physiological fluids with different ionic sizes, mobility, and lipophilicity (Table 4). The calculated selectivity coefficients (${K}_{OND, Int}^{pot}$) prove that interferences did not affect the performance of the working electrode and demonstrated the sensor capacity for plasma applications.

**Table 4:** Potentiometric selectivity coefficients of the optimized sensor for different interfering ions calculated using the separate solution method (59)

Interfering compound	${K}_{OND, Int}^{pot}$*
Potassium	4.33 × 10^−3^
Sodium	1.09 × 10^−3^
Ammonium	3.07 × 10^−3^
Calcium	2.73 × 10^−4^
Magnesium	5.30 × 10^−5^
Cadmium	1.20 × 10^−4^
ferrous	1.08 × 10^−4^
chromium	2.70 × 10^−4^

^*^Average of three determinations

The optimized sensor was successfully applied to determine OND in Zofran® tablet and spiked human plasma without prior extraction, pretreatment, or derivatization operations (Table 5).

**Table 5:** Statistical comparison of the developed potentiometric method to the reported HPLC method for determination of OND in Zofran® tablets form and plasma sample

Parameter	Zofran^®^ Tablet		Spiked human plasma samples	
	Developed potentiometric method	Reported^a^ HPLC method	Developed potentiometric method	Reported^a^ HPLC method (41)
Mean	100.01	100.84	98.26	98.67
SD	1.082	0.86	2.227	2.875
Variance	1.171	0.740	4.960	8.266
n	6	6	6	6
Student’s t-test^b^	1.471 (2.228)^b^		0.276 (2.228)^b^	
F value^b^	1.583 (7.146)^b^		1.667 (7.146)^b^	

^a^HPLC method using C18 column as the stationary phase and a mixture consisting of acetonitrile: 0.02 M sodium phosphate monobasic buffer (pH adjusted to 3.0 using phosphoric acid) in ratio (60:40, v/v) as a mobile phase. The mobile phase was pumped at a flowrate of 1.5 ml/min. UV detection was carried out at 305.0 nm[41]

^b^Values in parentheses are the corresponding tabulated two-tailed values at significance level α = 0.05

Results obtained for the analysis of OND were statistically compared with a reported HPLC method [41]. The calculated t and F values at a 95% confidence interval were less than the tabulated ones, revealing no significant difference between the developed and reported methods concerning accuracy and precision (Table 5).

Although the reported OND potentiometric sensor [46], showed a longer life span of 7 weeks, it sluggishly responded to OND (response time 18 s). The proposed sensor is based on a data-driven QbD approach based on a custom experimental design. Additionally, the sensor resulting from this approach expressed a relatively faster response for OND that suits real-time assay. Contrary to the reported sensor, the developed sensor is applicable for OND assay in plasma samples.

## Conclusions

The adopted quality by design approach employs a custom experimental design to develop and optimize a potentiometric sensor for OND assay. The sensor was designed to fit the desired analytical purpose. The sensor shows a Nernstian slope, high correlation coefficient, low quantification limit, and high selectivity for OND. The desirability function selected the optimal PVC membrane recipe to achieve optimal sensor performance. The potentiometric method was validated for OND assay in Zofran^®^ tablets and human plasma samples. The sensor can be used for fast, direct, sensitive, and selective OND assay in quality control laboratories, clinical laboratories, and pharmacokinetic studies.

## Data Availability

Datasets generated and/or analyzed during the current study are available from the corresponding author on reasonable request.
